# Birth Defects Associated with Prenatal Alcohol Exposure—A Review

**DOI:** 10.3390/children10050811

**Published:** 2023-04-29

**Authors:** Katarzyna Anna Dyląg, Florencia Anunziata, Gretchen Bandoli, Christina Chambers

**Affiliations:** 1 Department of Pathophysiology, Jagiellonian University Medical College, Czysta 18, 31-121 Krakow, Poland; katarzynaannadylag@gmail.com; 2 St. Louis Children Hospital, ul. Strzelecka 2, 31-503 Krakow, Poland; 3 Department of Pediatrics, University of California, San Diego, 9500 Gilman Drive, MC0828, La Jolla, CA 92093-0412, USA; fanunziata@health.ucsd.edu (F.A.); chchambers@health.ucsd.edu (C.C.)

**Keywords:** alcohol, major birth defects, review, pregnancy

## Abstract

Since the recognition of fetal alcohol syndrome, alcohol has been accepted as a human teratogen. However, little is known about the relation between prenatal alcohol exposure and the spectrum of associated major birth defects. The objective of this review was to summarize data on the association of major congenital abnormalities and prenatal alcohol exposure. We included all major birth defects according to ICD-10 classification. We found that the strongest evidence to date lies in the research examining herniation (gastroschisis and omphalocele), oral clefts (cleft lip with or without palate and cleft palate) and cardiac defects. There is less consistent evidence supporting the association between prenatal alcohol exposure and anomalies of gastrointestinal system, diaphragmatic hernia, genitourinary system and neural tube defects. We found no material support for PAE and choanal atresia, biliary atresia or clubfoot.

## 1. Introduction

Major birth defects result in biological, psychological, behavioral and lifestyle consequences which can lead to long-term disabilities and fetal death. They affect 3% of births in the United States [1], and approximately 3.2 million children per year worldwide [2]. Congenital defects are usually defined as structural or functional abnormalities occurring during gestation, at or before birth [3]. They could be syndromic (involving different organ systems) or isolated [4]. Although the causes of most major birth defects are unknown, there are several modifiable risk factors for specific birth defects, including prenatal alcohol exposure, a recognized teratogen.

The identification of an alcohol-related congenital syndrome (fetal alcohol syndrome, FAS) [5,6] have guided the literature on early developmental risks and prenatal alcohol exposure. Since that time, the teratogenic effect (ability to induce or increase the incidence of congenital malformations or neurobehavioral disorders) of alcohol has been widely studied [7,8,9,10,11,12,13,14,15,16]. According to the US National Institutes of Health (ncbi.nlm.nih.gov; October 2019), more than 17,000 articles arise linking the terms “fetal” and “alcohol”. In 1996, the Institute of Medicine (IOM) published the diagnostic criteria including a new umbrella term, fetal alcohol spectrum disorders (FASD). Four categories were described under this term: FAS, partial fetal alcohol syndrome (PFAS), alcohol-related neurodevelopmental disorder (ARND) and alcohol-related birth defect (ARBD). Most of the currently used diagnostic criteria follow the IOM categories; however, ARBD is not uniformly included [17,18,19]. In 2016, Hoyme et al. defined ARBD as one or more major malformations demonstrated in animal models and human studies to be a result of prenatal alcohol exposure [7]. The malformations categorized as ARBD by Hoyme et al. are: atrial septal defects, aberrant great vessels, ventricular septal defects, conotruncal heart defects, radioulnar synostosis, vertebral segmentation defects, large joint contractures, scoliosis, aplastic/hypoplastic/dysplastic kidneys and “horseshoe” kidneys/ureteral duplications. The defects of sensory organs are also included in this category (strabismus, ptosis, retinal vascular anomalies, optic nerve hypoplasia, conductive hearing loss and neurosensory hearing loss). According to these criteria, a child with an ARBD diagnosis does not necessarily have to present with central nervous system impairment. 

Although the evidence demonstrating the association between congenital abnormalities and maternal alcohol consumption is strong in animal models, the data on human models are dispersed and less obvious. Research has shown that prenatal alcohol exposure can affect the organ systems in several ways [4], including through free radical production, by promoting cell death, through disrupted cell migration and by interference with growth factor functions [20]. These mechanisms may result in PAE-associated congenital anomalies that fall outside of the traditionally defined ARBD, which may escape the attention of clinical providers. 

The objective of this review was to summarize evidence from studies examining the association between periconceptional exposure to alcohol and major congenital abnormalities, grouped by major organ system (ICD-10) classification. The discussion focuses on the overall strength of the evidence, study design and quality and future directions.

## 2. Methods

Studies reporting congenital birth defects and prenatal alcohol exposure were included in the review. Only publications in English were included. The abstracts were screened by the main researcher (KD) with the use of Abstrackr^®^ [21]. 

### Search Strategy

The search procedure was determined before the search was performed. The PubMed database was used to identify potentially relevant publications. All papers were included up to 2021, when the search was conducted. Terms: “prenatal alcohol exposure”, “fetal alcohol spectrum disorders”, “fetal alcohol birth defects”, “alcohol intake”, “alcohol consumption” and “alcohol drinking” were employed to identify research that included maternal alcohol consumption during pregnancy. Each PAE search string was combined with the major malformation groupings, with the following resulting papers: cardiac birth defects (number of resulting papers = 307): “congenital cardiac defect”, “congenital heart anomaly”, “common arterial trunk”, “ventricular septal defect”, “atrial septal defect”, “tetralogy of Fallot”, “pulmonary valve atresia”, “pulmonary valve stenosis”, “pulmonary valve insufficiency”, “Ebstein’s anomaly”, “hypoplastic right heart syndrome”, “stenosis of aortic valve” “insufficiency of aortic valve”, “mitral stenosis”, “mitral insufficiency”, “hypoplastic left heart syndrome”, “dextrocardia”, “patent ductus arteriosus”, “coarctation of aorta”, “interruption of aortic arch”, “congenital tricuspid stenosis”. Renal defects (n = 387): “kidney defect”, “horseshoe kidney”, “kidney aplasia”, “kidney hypoplasia”, “ectopic kidney”, “renal dysplasia”, “kidney dysplasia”, “hydronephrosis”, “polycystic kidney”, “renal cyst”, “megaureter”, “renal anomalies”. Oral clefts (n = 164): “cleft palate”, “cleft lip”, “orofacial clefts”, “oral clefts”. Gastrointestinal system defects (n = 477): “esophageal atresia”, “atresia of esophagus”, “pyloric stenosis”, “malrotation”, “anal atresia”, “Hirschsprung disease”, “billiary atresia”, “liver malformation”, “spleen malformation”, “pancreas malformation”. Defects of herniation (n = 189): “omphalocele”, “omphalocoele”, “gastroschisis”, “diaphragmatic hernia”, “inguinal hernia”, “umbilical hernia”, “herniation”. Bones and spine defects (n = 158): “pectus excavatum”, “pigeon chest”, “pectus”, “spina bifida”, “congenital dislocation of hip”, “congenital coxa valga”, “congenital coxa vara”, “skeletal anomalies”, “pes cavus”, “club foot”, “congenital talipes equinovarus”, “cavoid foot”, “scoliosis”. Genitourinary tract defects (n = 230): “agenesis of uterus”, “aplasia of uterus”, “hypoplasia of uterus”, “Imperforate hymen”, “fusion of labia”, “cryptorchidism”, “undescended testicle”, “hypospadias”, “hypoplasia of penis”, “micropenis”, “genitourinary tract malformation”. Anomalies of nose and larynx (n = 19): “laryngomalacia”, “choanal atresia”, “laryngeal web”, “laryngeal hypoplasia”. 

After the initial search, papers were evaluated for duplicates and English language. Papers that did not quantify an effect estimate of PAE and the select malformation were then removed. One hundred and four articles remained after these screening criteria were applied. We further removed papers that did not provide adjusted effect estimates of PAE and major malformations, as unadjusted effect estimates were likely biased. This final exclusion resulted in 58 papers synthesized in the review. 

The 58 papers included in the review were grouped by major system (ICD chapter), and were presented by year, study population, study design, number of cases, how prenatal alcohol exposure (PAE) was operationalized (e.g., drinks per week), how and when PAE was collected (e.g., self-report about first trimester), covariates included in models and the findings (Supplementary Tables S1–S7). We then summarized each table by study design (case control, cohort or cross-sectional), the proportion that limited exposure to the periconceptional period (defined here as analyses limited to any timeframe from the three months before conception to three months post-conception, representing the critical period in organogenesis), the proportion of all studies with significant findings (*p* < 0.05) and the proportion with significant findings among those that queried PAE during the periconceptional period (Table 1). 

## 3. Results

In total, 58 unique studies reported results on cardiac, urinary, oral cleft, gastrointestinal, hernia, skeletal and genital defects (Table 1). Cardiac defects, oral clefts and hernia had the largest literature reviewed, and tended to have the most support for an association between PAE and the defect. Urinary, gastrointestinal and genital defects had less supporting evidence reviewed, while skeletal defects were unsupported in this review. The details of each malformation grouping follow.

### 3.1. Cardiac Defects

There were 17 unique studies that evaluated PAE and cardiac defects. The majority were case controls (n = 11), followed by cohort (n = 4) and cross-sectional (n = 2) (Supplementary Table S1). 

#### 3.1.1. Any Cardiac Defects

There were 11 studies that evaluated “any cardiac defect” [22,23,24,25,26,27,28,29,30,31,32]. Half of the studies (n = 5) queried specifically about alcohol use in first trimester or periconceptional period [22,23,24,26,28]. Of these studies, only one found evidence of an association between PAE in the first trimester and any cardiac defects. Mateja et al. [28] found associations between maternal binge drinking prior to pregnancy and the risk of congenital heart defects (OR = 2.9 (95% CI 1.1, 7.5)). This relationship was stronger if combined with maternal smoking. The other studies were null. For example, in a large study in 2008 using data from the National Birth Defects Prevention study, Malik [26] found no association between PAE (yes/no) in the time around conception and cardiac defects (OR 0.9, 95% CI 0.8, 1.0). There were a handful of studies that reported positive associations, but did not limit PAE to the critical period. For example, in a Canadian cohort, Liu et al. [31] reported that maternal alcohol and other substance use at any time during pregnancy was significantly associated with the risk of congenital heart defects in the offspring (OR 1.9, 95% CI 1.7, 2.0). Stronger results were found by Pei et al. [30] when evaluating >1 drink/week and cardiac defects (prevalence ratio 3.2, 95% CI 1.0, 10.1), but again, alcohol was assessed as any use during pregnancy. 

#### 3.1.2. Ventricular Septal Defect

Four studies evaluated ventricular septal defects (VSD) [33,34,35,36], for which two limited PAE to the periconceptional period [33,34]. Williams et al. did find evidence of an association at the highest levels of PAE (10+ drinks per week: OR 3.1, 95% CI 1.2–8.2) using a case-control design from the Metropolitan Atlanta Congenital Defects Program (MACDP) [33]. In contrast, Strandberg-Larsen [34] found no evidence of an association between PAE and VSD from a Danish birth cohort, although confidence intervals were wide. Strandberg-Larsen evaluated PAE both by drinks per week and binge episodes, and although some estimates suggested a relationship (e.g., −3+ binge episodes prevalence ratio 1.3, 95% CI 0.7, 2.4), all confidence intervals largely overlapped the null. Although the study did not limit the exposure window to the periconceptional period, O’Leary [35] found an increased risk of VSDs in children of mothers diagnosed with an “alcohol-related diagnosis” during pregnancy (OR: 2.1 (95% CI 1.3, 3.4)). 

#### 3.1.3. Atrial Septal Defect

One study looked specifically at atrial septal defects (ASD). Strandberg-Larsen et al. [34] evaluated both binge consumption and drinks per week and did not find any associations with ASDs.

#### 3.1.4. Conotruncal Heart Defects

Two studies evaluated conotruncal defects, of which one classified PAE in the periconceptional period [37,38]. In a case-control study in 2003, Carmichael [37] found slight increased odds of one or more drinks per week (OR 1.6, 95% CI 0.8, 3.0) among mothers of children born with conotruncal defects, but confidence intervals were very wide and overlapped the null. Separately, a study utilizing California birth records found an association between conotruncal congenital heart disease and maternal alcohol-related ICD diagnosis during pregnancy (RR 1.6, 95% CI 1.2, 1.8) [38].

#### 3.1.5. Other Heart Defects

No adjusted studies were found on the coarctation of the aorta [22] or single ventricle [39]. Moreover, no studies on other heart defects such as Ebstein’s anomaly, stenosis or insufficiency of heart valves, dextrocardia, patent ductus arteriosus or interruption of aortic arch were identified in this review process. 

### 3.2. Urinary System Defects

Seven studies were found to evaluate PAE and urinary defects (hydronephrosis, renal agenesis, renal anomalies) [23,25,35,40,41,42,43]. The majority were case controls (n = 6) and one was a cohort study (Supplementary Table S2). More than half of the studies (n = 4) evaluated alcohol exposure during the periconception period [23,40,42,43]. Moore et al. reported a small association between all renal anomalies and 3–13 drinks/week (OR 1.5, 95% CI 1.0, 2.3). In that study, most associations with specific urinary defects were null, with the exception of one positive association between PAE and renal agenesis/hypoplasia (OR = 2.5, 95% CI 1.2, 5.1 for 3–13 drinks/week), and a positive association between <3 drinks per week and multicystic kidney dysplasia (OR 2.4, 95% CI 1.0, 5.6) [40]. A case-control study by Parikh et al. [41] found an association between renal agenesis and PAE (OR 2.3, 95% CI 1.0, 5.4), and a small case-control study from the National Birth Defect Prevention Study [42] found an association between binge PAE and bilateral renal agenesis or hypoplasia (OR 1.9, 95% CI 0.9, 4.2), although both studies had confidence intervals that included the null. There was only one study that specifically evaluated hydronephrosis and maternal alcohol-related diagnoses, and reported a positive association (OR = 2.1, 95% CI 1.3, 5.3) [35]. No evidence of an association between urinary anomalies and PAE was found in the remaining studies reviewed [23,25,43], regardless of the levels of alcohol exposure.

### 3.3. Oral Clefts

Oral clefts (OC) are one of the most studied malformations in relation to prenatal alcohol exposure (Supplementary Table S3). The embryology of cleft lip (CL), cleft lip with or without cleft palate (CLP) and cleft palate alone (CP) suggests the teratogenic potential of alcohol exposure during this sensitive stage in development [44]. 

There were 16 studies evaluating OCs and PAE [23,25,30,32,45,46,47,48,49,50,51,52,53,54,55,56]. The majority were case-control studies (n = 13), two were cohort studies and one was cross-sectional. Of these studies, 75% (n = 12) queried specifically about alcohol use during the critical period (periconception) [23,45,46,47,48,49,50,51,52,53,54,55]. Half of the studies reported significant associations between PAE and OCs, and almost all of them limited PAE to the periconceptional period (44%). 

#### 3.3.1. All Oral Clefts

Four studies evaluated all OCs combined, of which two assessed alcohol use during the periconceptional period. Only Pei et al. (2015) found an association between OCs and having a history of alcohol exposure during gestation (prevalence rate ratios = 9.0, 95% CI 2.0, 39.1) [30]. However, this result should be viewed cautiously, as alcohol exposure was not limited to the critical period and the confidence interval is wide. The remaining studies reported null results. For example, Werler et al. [45] used a case-control design in an American/Canadian population examining alcohol use during the first four lunar months of pregnancy and found no associations between OCs combined and 5+ drinks/day (OR = 1.8, 95% CI 0.8, 4.4). In a Danish case-control study, Bille et al. [53] found no association between alcohol consumption (yes/no) during the first trimester of pregnancy and all OCs combined (OR = 1.1, 95% CI 0.8, 1.5). Finally, a Spanish study [25] which did not limit alcohol use to the critical period also found no association between different levels of PAE and all OCs combined, although at the highest level of alcohol, the effect estimate only slightly crossed the null (OR = 4.5, 95% CI 0.9, 30.1 for >92 gm daily ingestion).

#### 3.3.2. Cleft Lip with or without Cleft Palate or Cleft Palate Only

There were handful of studies evaluating the association of prenatal alcohol exposure and CLP or CP alone (n = 14). From these studies, 12 assessed alcohol consumption during the critical period in gestation. Seven reported significant results and all of them limited alcohol use to the periconceptional period. For example, Lorente et al. [48] found that alcohol consumption in pregnancy was a risk factor for CP only (OR = 2.2, 95% CI 1.0, 5.1). Shaw et al. [47] showed an increased risk of isolated CLP for drinking >5 drinks/day weekly or more frequently (OR = 3.4, 95% CI 1.1, 9.7), and of multiple CLP for the same level of prenatal alcohol exposure (OR = 4.6, 95% CI 1.2, 18.8). A case-control study analyzing a Brazilian population showed significant associations between CLP and CP and drinking more than 96 gm of alcohol per day during the first trimester of gestation (OR = 2.1, 95% CI 1.3, 3.4 and OR = 2.9, 95% CI 1.3, 8.3, respectively) [55]. Munger et al. found that only exceeding 10 drinks/month was significant for CLP (OR 4.0 95% CI 1.1, 15.1) [46]. Three studies found that women who reported binge drinking (>5 drinks/day) were more likely to give birth to a child with CLP [45,47,54]. Finally, in a small case-control study, Beaty et al., 2001, found CLP to be less common among children of mothers who reported any alcohol intake in pregnancy (OR = 0.4, 95% CI 0.2, 0.8) [49]. 

Three studies attempted to assess a dose response or threshold dose. However, none of these studies demonstrated an increased trend for OCs according to the mother’s alcohol intake [23,50,52].

### 3.4. Anomalies of Gastrointestinal Tract

Very few studies (n = 4) documented the impact of alcohol consumption during pregnancy on the fetal gastrointestinal system (Supplementary Table S4) apart from unadjusted associations. Among those studies with multivariable analysis included in this review, three were case-control studies and one was a cohort study. Half of the studies (n = 2) assessed alcohol exposure during the periconceptional period. 

#### 3.4.1. Esophageal Atresia

Three studies evaluated esophageal atresia, for which one showed increased odds for binge drinking during the first trimester of pregnancy (OR = 2.6, 95% CI 1.0, 6.6) [57]. In a separate study using data from the National Birth Defects Prevention study in the U.S. [58], there was no association between any periconceptional alcohol exposure and this defect (OR = 1.2, 95% CI 0.8, 1.8). 

#### 3.4.2. Gastrointestinal Obstruction

Only one study evaluated gastrointestinal obstruction as an outcome. In a cohort study from Japan, no association between gastrointestinal obstruction and PAE was found using a cohort design [32] (OR = 1.3, 95% CI 0.7, 2.4 for quitting during early pregnancy; 1.7, 95% CI 0.4, 7.6 for current drinkers). 

#### 3.4.3. Intestinal Atresia/Anal Atresia

There was only one study evaluating intestinal atresia/anal atresia [25]; however, it reported null results for different alcohol doses. This Spanish study assessed alcohol consumption anytime during pregnancy and asked about the type of alcoholic beverages, the amount of alcohol consumed per day and the specific gestational week of exposure (OR = 1.2, 95% CI 0.8, 1.8 for <10–20 gm sporadically during gestation; OR 1.3 95% CI 0.2, 7.5 for >90gm or sporadic binges during gestation; 0.9, 95% CI 0.6, 1.4 for 16–48 gm daily ingestion; 1.7, 95% CI 0.3, 8.9 for 56–88 gm daily range; and 3.0, 95% CI 0.5, 21.6 for >92 gm daily ingestion).

Despite several anecdotal reports [59,60,61], no study examining the association of pyloric stenosis and prenatal alcohol exposure was found for in this review. Furthermore, no studies on liver malformations, pancreas malformations, Hirschsprung disease or malrotation were encountered using the previously defined search and evaluation criteria.

### 3.5. Herniation Defects

Herniation is one of the most studied congenital malformations, and has the most evidence in support of an association with PAE (57% positive results) (Supplementary Table S5). In total, 12 studies evaluated herniation and PAE and all of them used case-control designs. The majority classified PAE in the periconceptional period (83%) [62,63,64,65,66,67,68,69,70,71]. Details of the findings are as follows:

#### 3.5.1. Diaphragmatic Hernia

There were three studies that evaluated diaphragmatic hernias (Supplementary Table S5). Two queried about alcohol use before and in early pregnancy [62,63], although results from both studies, which also assessed exposure by quantity, were null. In a third study that assessed any report of alcohol use in pregnancy, PAE conferred a 3.5-fold increase in odds of diaphragmatic hernia (OR = 3.6, 95% CI 1.4, 9.8) [72].

#### 3.5.2. Gastroschisis

We identified nine studies that examined the odds for gastroschisis and PAE [64,66,67,68,69,70,71,73,74]. There was evidence of a positive association between PAE and gastroschisis in 6 of the 9 papers [64,68,69,70,71,74] with the odds ratio ranging from 1.38 [71] to 15.1 [70]. The two studies that did not find an association between binge PAE and gastroschisis were both from United Kingdom [66,67], and although ORs were consistent with the other studies, confidence intervals were quite wide (OR = 1.5, 95% CI 0.6–3.5; OR = 1.6, 95% CI 0.6–4.2, respectively).

Richardson et al. [74] used data from the U.S. National Birth Defect Prevention Study between 1997 and 2005 and found an increased association between binge drinking during the critical period and gastroschisis (OR = 1.5, 95% CI 1.2, 1.9). This study also had positive findings for 1–4 drinks/month (OR = 1.3, 95% CI 1.0, 1.6); 5–15 drinks/month (OR = 1.5, 95% CI 1.1, 2.0); more than 30 drinks/month (OR = 1.9, 95% CI 1.3, 2.7); and for drinking that did not constitute binge drinking (OR = 1.3, 95% CI 1.0, 1.6). Additionally, Werler et al. [64] reported an increased risk for gastroschisis in children born to mothers who consumed >6 drinks/week (OR = 2.9, 95% CI 1.1, 7.4) and >5 drinks/any time (OR = 3.2, 95% CI 1.5, 6.7) in data from a Slone Birth Defects study.

#### 3.5.3. Omphalocele

Two large studies from the U.S. National Birth Defects Prevention Study found increased odds of omphalocele in children prenatally exposed to alcohol [71,74]. Both studies found positive associations with any alcohol use during the periconceptional period (OR = 1.5, 95% CI 1.0, 2.2, Bird et al., 2009, and OR = 1.5, 95% CI 1.1, 2.0, Richardson et al., 2011). Richardson et al. also reported significant odds for 1–4 drinks/month (OR = 1.5, 95% CI 1.1, 2.1); intake of 16–30 drinks/month (OR = 1.7, 95% CI 1.0, 2.9); drinking but not binge drinking (OR = 1.4, 95% CI 1.1, 1.9); and binge drinking (OR = 1.7, 95% CI 1.2, 2.4) [74].

### 3.6. Neural Tube Defects and Skeletal Defects

Although etiologically different, neural tube defects and skeletal defects were combined due to the small sample (n = 6, Supplementary Table S6). All of them used case-control designs, limited their alcohol assessment to the periconceptional period and reported null results (Table 1).

#### Clubfoot, Spina Bifida and Neural Tube Defects

There was no evidence of an increased risk for clubfoot (ORs = 1.1, 95% CI 0.8, 1.5 for <3 drinks/day and 1.2, 95% CI 0.8, 1.9 for >3 drinks/day) [75], spina bifida [76,77] or neural tube defects, regardless the amount and frequency of alcohol consumption during pregnancy [23,78]. Inverse associations ranging from ORs of 0.6 to 0.8 were observed by Louden et al. for any alcohol consumption, binge drinking and drinks/month and all neural tube defects combined [79]. Most confidence intervals for these associations excluded the null.

### 3.7. Anomalies of the Genitals

Seven studies evaluated genital defects and PAE (Table 1). The majority were case controls (n = 5), followed by cohort (n = 2). Some of them (n = 2) limited their alcohol assessment to the periconceptional period [80,81]. Most of the studies did not find an association between PAE and any genital anomalies (70%). Two found significant findings, of which only one assessed alcohol during the critical period in gestation [80].

#### 3.7.1. Cryptorchidism

Five studies looked specifically at cryptorchidism [80,81,82,83,84], but only two queried about alcohol use in the periconceptional period (Supplementary Table S7). From these two, a study conducted in Italy [80] found an association between drinking >8 drinks/week and cryptorchidism (OR = 4.6, 95% CI 1.2, 16.8). The other study that queried alcohol use during the critical period was conducted in Denmark [81]. They did not find an association between this genital birth defect and PAE from any number of binge drinking episodes.

A large Danish–Finish study analyzed drinks per week and the risk of cryptorchidism [83]. They found an OR of 1.2 (95% CI 1.0, 1.5) when analyzing PAE as a continuous variable, and found effects at over 4 drinks per week when looking at individual quantities per week. The other three studies evaluating cryptorchidism and PAE but not limiting alcohol use to periconception were null.

#### 3.7.2. Hypospadias

The evidence of an association between hypospadias and maternal drinking in our review was weak. We only identified one study, in which the authors found a positive association between 8+ drinks per week and hypospadias, but confidence intervals were very wide and consistent with null results [80].

#### 3.7.3. Genital Defects

Two studies analyzed any genital defects. The results of these studies, conducted by Martinez-Frias et al. [25] and Kurita et al. [32], did not report an association between genital defects and any amount or frequency of alcohol consumption during pregnancy.

### 3.8. Other Anomalies

Kancherla et al. found no association between choanal atresia in infants and prenatal alcohol consumption (OR = 1.0, 95% CI 0.7, 1.5) or maternal binge drinking in pregnancy (OR = 1.2, 95% CI, 0.8, 1.8) [85]. There were no studies documenting laryngomalacia, laryngeal web or laryngeal hypoplasia in association with PAE.

## 4. Discussion

In this review, we attempted to review the evidence on the association between congenital malformations and prenatal alcohol exposure. Unlike previous work, we have not limited the review to malformations previously suggested to be possible ARBDs, but instead synthesized the evidence for all studies identified that presented multivariable adjustment for prenatal alcohol in relation to major malformations, summarized by major organ system/ICD grouping. Looking across birth defects, the highest volume of scholarship was focused on the cardiac system, oral clefts and herniation, each of which also had the most evidence in support of associations. More limited research was available on genital, urinary, skeletal or gastrointestinal defects. However, within those studies, urinary and genital defects had some evidence supporting associations, while skeletal and gastrointestinal studies had weak to no evidence supporting associations with PAE.

While many of the studies reviewed in this work are included in the classification of ARBD designation [7] (which includes cardiac: atrial septal defects, aberrant great vessels, ventricular septal defects and conotruncal heart defects; skeletal: radioulnar synostosis, vertebral segmentation defects, large joint contractures and scoliosis; renal: aplastic/hypoplastic/dysplastic kidneys and “horseshoe” kidneys/ureteral duplications; eyes: strabismus, ptosis, retinal vascular anomalies and optic nerve hypoplasia), others were not. The strongest evidence in our review for malformations outside of ARBD was for oral clefts and for herniation defects, particularly gastroschisis. The intent of including malformations outside of the traditional ARBD classification was to urge others to weigh the evidence and consider whether a causal etiology exists between PAE and these malformations.

Congenital malformations are usually multifactorial and rare, making the identification of single risk factors difficult. Although the majority of the studies included in the review were well-designed case-control studies performed with the use of birth defect registers, there are numerous factors that could potentially affect the results reported by the authors [86]. First, information about PAE was collected retrospectively, with the period of recall often extending back several years. We noted the heterogeneity of how the information about PAE was collected and quantified. The authors defined the quantity of alcohol consumption by drinks per drinking days [45], drinks per occasion [47], drinks per week [23], drinks per month [46] or with estimated intake in grams [25]. The type of consumption was categorized as binge/not binge [74] or sporadic/regular [25]. The timing of exposure was mostly classified as before/after the pregnancy was recognized [87], or in/after the first 12 weeks of pregnancy [66]. However, there were some articles that did not limit alcohol exposure to the periconceptual period [25,29,30,35,36]. These differences may have led to inconsistency across studies, and limited the ability to compare results between them, as PAE limited to exposure after the critical period would essentially be misclassified, attenuating results. Moreover, there were many potential confounders that could have influenced the results. We restricted the sample of articles to those that published adjusted estimates, although there was wide variation in what variables were included in analysis, which could have led to biased results. Even though the authors adjusted for potential confounders (maternal age, marital status, socioeconomic status, parental education, smoking, etc.), there are other factors that often were not included in the analyses, including maternal nutrition, co-exposures, comorbidities or contact with other teratogenic substances. We caution overinterpretation of estimates from studies that adjusted for only one or two covariates. For this reason, we noted which variables were used for analytic adjustment in the tables.

The major strength of our review was the comprehensive approach and selection criteria. To our knowledge, this was the first review to include articles looking at selected birth defects and various levels and gestational timing of exposures to alcohol, which may be more generalizable than studies focusing only on offspring with FASD. In addition, we attempted to expand beyond malformations previously typically associated with PAE. Finally, we required multivariable adjustment, as unadjusted estimates are highly likely to be confounded. A limitation of the review was that only studies in English and one database were included in the searching process, although only one non-English language paper was excluded that would have otherwise met criteria. Additionally, as previously discussed, effect estimates may have remained confounded despite multivariable adjustment, and some estimates may have been attenuated due to assessing PAE only after the critical period. Furthermore, there were differences in how the periconceptional period was defined across papers, though all exposure windows likely included the critical organogenesis period. Moreover, it is possible that this review may not have covered all articles discussing ARBD malformations, as it only reports research that satisfied search criteria such as study design and multivariable adjusted estimates. Consequently, it should not be concluded that this is an exhaustive list of malformations studied for alcohol sensitivity. Finally, publication bias may have played a role if positive studies are more likely to have been submitted or published, which may have resulted in an overestimate using the available evidence.

## 5. Conclusions

In conclusion, our review supports the role of alcohol as a human teratogen across multiple organ systems. However, it is important for clinicians not to emphasize the association with PAE when providing care for individual patients with birth defects to avoid stigma. Based on the biologic possibility and inconsistent evidence of the association between PAE and congenital abnormalities, studies on birth defects, ideally with prospective data on maternal alcohol intake and robust measure of confounding factors, should be prioritized.

## Figures and Tables

**Table 1:** Summary of results.

Birth Defect	Type of Study			Number of Studies ^a^	Significant Findings from All Studies (n, (%))	Timing of Alcohol Exposure Assessment (n, (%))	Significant Findings Among Papers with Periconceptional Exposure (n, (%))
	Case Control	Cohort	Cross Sectional		*p* = 0.05	Periconceptional-First Trimester	*p* = 0.05
Heart	11	4	2	17	8 (47%)	9 (53%)	2 (22%)
Urinary	6	1	0	7	2 (28%)	4 (57%)	1 (25%)
Oral clefts	13	2	1	16	8 (50%)	12 (75%)	7 (58%)
Gastrointestinal	3	1	0	4	1 (25%)	2 (50%)	1 (50%)
Hernia	12	0	0	12	8 (58%)	10 (83%)	6 (60%)
Skeletal	6	0	0	6	0 (0%)	6 (100%)	0 (0%)
Genital	5	2	0	7	2 (29%)	2 (29%)	2 (100%)

^a^ The number of studies does not sum the total number of articles (n = 58) because some included articles published on more than one birth defect.

## Data Availability

Not applicable.

## Supplementary Materials

The following supporting information can be downloaded at: https://www.mdpi.com/article/10.3390/children10050811/s1, Tables S1–S7: Studies of prenatal alcohol exposure and the risk of cardiac defects.

## Abbreviations

ARBD	Alcohol-related birth defect
ARND	Alcohol-related neurodevelopmental disorders
FASD	Fetal alcohol spectrum disorders
FAS	Fetal alcohol syndrome
PAE	Prenatal alcohol exposure
PFAS	Partial fetal alcohol syndrome
