# Supplementary material for: Birth Defects Associated with Prenatal Alcohol Exposure—A Review
Source: Children (Basel). 2023 Apr 29;10(5):811. doi: 10.3390/children10050811 (PMC10217313; doi:10.3390/children10050811)
Supplement: Supplementary file 1 [file children-10-00811-s001.zip › children-2354967-supplementary.pdf]

Supplementary Materials

Suppl. Table S1. Studies of prenatal alcohol exposure and the risk of cardiac defects

Birth defect	Author	Year	Study population	Study design	Number of cases	Prenatal Alcohol Exposure	Prenatal alcohol exposure measurement	Covariates	Findings
Heart defects	Tikkanen et al.	1991	Finnish	Case-control	N=573	Alcohol consumption yes/no	Interviewed by midwives using a structured questionnaire at maternity welfare centers - 92-96 days after delivery, about alcohol consumption during the first gestational trimester.	Maternal age	OR 1.2 (95% CI 0.7, 2.4) for any alcohol intake in the first trimester
Heart defects	McDonald et al.	1992	American/Canadian	Case-control	N=318, controls: healthy children	Seven or more drinks per week	Survey of occupational factors in pregnancy conducted in Montreal from 1982 to 1984. Interviews about alcohol consumption during the first trimester of pregnancy.	Maternal age, educational level, and ethnic (color and language), cigarette and coffee consumption	OR = 0.91 (0.7, 1.2) for 1-2 drinks per week; OR= 0.96 (0.6, 1.5) for 3 to 6 drinks per week; OR= 1.24 (0.7, 2.2) for 7< per week.
Heart defects	Cedergren et al.	2002	Swedish	Case-control	N=227	Alcohol use: yes, no, not stated.	Data from medical records and from the Medical Birth Register prior the knowledge of the outcome. Mother's first visit to the antenatal care center (usually week 10–12 of pregnancy) and asked about alcohol consumption during early pregnancy.	Maternal age	OR =0.95 (0.69, 1.31) for alcohol use (adjusted by matching)
Heart defects	Martinez-Frias et al.	2004	Spanish	Case-control	N=204	Type of alcoholic beverages, the amount per day (or other specified periodicity), and the moments of pregnancy. Standard drink of beer or wine = 10 gm and of distilled	Interview the mothers within the first three days after delivery about alcohol consumption during all the gestational period	Birth weight, length, and occipital-frontal circumference, smoke during pregnancy	OR=1.01 (0.8, 1.3) for <10-20gm sporadically during gestation; OR=1.7 (0.4, 8.7) for >90gm or sporadic binges during gestation; OR=0.8 (0.6, 1.2)

						spirits has 20 gm.			for 16-48gm daily ingestion; OR=2.0(0.3, 15.6) 56-88gm daily range ingestion; OR=11.9 (1.6, 2.5) for > 92gm daily ingestion.
Heart defects	Malik et al.	2008	American	Case-control	N=3067	Maternal alcohol use: yes, or no. Alcohol consumption as a covariate.	Data from the National Birth Defects Prevention Study 1997-2022. Extensive interview during pregnancy and the 1st year of life about alcohol consumption during 1 month before conception through 3 months after conception.	Residence of mothers	OR=0.9 (0.9, 1.0) for maternal alcohol use.
Heart defects	Hobbs et al.	2011	American	Case-control	N=417	Yes, No. Alcohol user: drinks per week 0.5 (0.2–2.0)	Arkansas Reproductive Health Monitoring System, a statewide birth defects registry and the National Birth Defects Prevention Study. Maternal interview and home visit data about alcohol consumption during pregnancy.	Age, race, educational level, household income, multivitamin supplement intake, number of cigarettes smoked daily and body mass index.	OR=0.9 (0.8, 1.0) for PAE.
Heart defects	Mateja et al.	2012	American	Case-control	N=237	Any alcohol use= 1 drink (14 g)/ week. Frequent drinking= average of 7 drinks/week. Binge= 5 or more drinks/occasion. Binge more than once= 1 Binge drinking or more, more than once in the 3 months.	Pregnancy Risk Assessment Monitoring Survey. Surveys are typically completed 3–6 months after birth about alcohol consumption 3 months prior to pregnancy (early pregnancy/conception).	Smoking, infant gender, maternal age, race, ethnicity, marital status, insurance and socioeconomic status, maternal psychosocial variables (stress).	OR=3.0 (1.2, 7.5)[a] binge drinking >1 time 3 months prior to pregnancy

Heart defects	Liu et al.	2013	Canadian	Cohort	N=2 278 838	Yes or no during pregnancy.	Canadian Institute for Health Information, birth records of all pregnancy and alcohol consumption during pregnancy.	Maternal age, parity, residence, multiple gestation, infant sex, region and year of birth.	OR= 1.9 (1.7, 2.0) for PAE.
Heart defects	Pei et al.	2015	Chinese	Cross-sectional	N=29098 infants screened	Alcohol intake frequency during pregnancy: No, <1/week, 1/week.	Structured questionnaire and medical records from local Hospitals about alcohol consumption during pregnancy.	Infant gender, fetal number, residence during the pregnancy, socio economic status, mother's education, mother's age, and parity.	Prevalence rate ratios: 1.1 (0.3, 4.3) alcohol intake <1 drink/week; 3.2 (1.0, 10.2) alcohol intake ≥1/week
Heart defects	Liu et al.	2020	Chinese	Cross-sectional	N= 29,098	Alcohol intake frequency: no, <1/week; ≥1/week	Large-scale epidemiologic survey/interview of birth defects, family and reproductive history about alcohol consumption during pregnancy.	Geographic maternal residence during pregnancy	OR= 1.2 (0.3, 5.1) for <1 week; 0.9 (0.1, 6.9) ≥1/week
Heart defects	Kurita et al.	2021	Japanese	Cohort	N=799	Nondrinkers: no alcohol consumption and/or quit drinking before pregnancy. Early drinkers: quit drinking during early pregnancy. Currently drinking: until the 2nd and 3rd trimester. Low (<1.5 drinks/week) and high (1.5+ drinks/week) absolute alcohol amount.	Self-reported questionnaires during the 2nd and 3rd gestational trimester and data from the Japan Environment and Children's Study. 1) no alcohol consumption, (2) quit drinking before pregnancy, (3) quit drinking during early pregnancy, and (4) currently drinking during the 2 and 3 trimester.	Maternal age, smoking habit of the mothers, mother medical and obstetric history, maternal body mass before pregnancy, pregnancy complications.	OR= 0.9 (0.7, 1.0) for quit drinking during early pregnancy; 0.8 (0.5, 1.2) for current drinker.
Ventricular septal defect	Williams et al.	2004	American	Case-control	N=122	None, light (five drinks per week or less), moderate (five to nine drinks per week) and	Data from the Atlanta Birth Defects Case–Control Study. Standardized interviews/ maternal self-report. Three	Maternal age, multivitamin use, maternal overt diabetes and race	OR=1.0 (0.7, 1.4) for 1-4 drinks/week; 1.2 (0.3, 3.4) for 5-9

						heavy (>10 drinks per week). Frequency of binge drinking, five or more drinks per sitting: none, less than once per week, and more than once per week. First trimester.	months prior to pregnancy through the first trimester.		drinks/week and 3.1 (1.2, 8.2) for heavy alcohol intake ≥ 10 drinks/week. Binge drinking OR=1.1 (0.6, 1.8) for less than once/week; 1.9 (0.7, 4.7) for more than once/week.
Ventricular septal defect	Strandberg-Larsen et al.	2011	Danish	Cohort	N=198	Average weekly intake. (1 drink 12g). Nondrinkers, and drinkers of: ½ -1½, 2–2½, and 3 or more drinks per week. Binge drinking in the periods between 16 and 31 gestational weeks.	Maternal interviews at 16 and 31 gestational weeks about alcohol consumption during the 1st and 2nd gestational trimester.	Maternal age, household socio-occupational status, parity, time to pregnancy in months, and smoking during pregnancy.	Prevalence rate ratios: 1.2 (0.9, 1.7) for ½ -1½ drinks per week; 1.4 (0.8, 2.3) for 2 drinks per week; 1.1 (0.5, 2.2) for 3+ drinks per week. Binge drinking PR: 0.9 (0.6, 1.3) for one; 1.1 (0.6, 1.9) for two; 1.3 (0.7, 2.5) for 3+.
Ventricular septal defect	O'Leary et al.	2013	Australian	Cohort	N=1365	Maternal alcohol diagnoses during pregnancy: (1) mental and behavioral disorder (alcohol-related), (2) alcohol-related diseases with a 100% attributable fraction (e.g., alcoholic liver disease), and (3) other alcohol codes.	Western Australian Data Linkage Unit, data collected from birth records about alcohol consumption during pregnancy.	Maternal age and year of birth, maternal Aboriginal status, maternal illicit drug use and socioeconomic status	OR: 2.2 (1.3, 3.4) for maternal alcohol related diagnoses.

Ventricular septal defect	Kovalenko et al.	2018	Russian	Case-control	N=233	Yes or no during pregnancy.	Murmansk County Birth Registry about alcohol during pregnancy.	Birth weight, baby sex, maternal age at delivery and body mass index, multivitamins and folic acid intake during pregnancy, smoking and drug abuse during pregnancy, maternal diabetes	OR = 4.8 (1.9, 12.4) for alcohol use
Atrial septal defect	Strandberg-Larsen et al.	2011	Danish	Case-control	N=145	Average weekly intake. (1 drink 12g). Nondrinkers, and drinkers of: ½ -1½, 2–2½, and 3 or more drinks per week. Binge drinking in the periods between 16 and 31 gestational weeks.	Maternal interviews at 16 and 31 gestational weeks about alcohol consumption during the 1st and 2nd gestational trimester.	Maternal age, household socio-occupational status, parity, time to pregnancy in months, and smoking during pregnancy.	Prevalence rate ratios: 1.0 (0.7, 1.5) for ½ -1½ drinks per week; 0.5 (0.2, 1.1) for 2 drinks per week; 0.7 (0.3, 1.6) for 3+ drinks per week. Binge drinking PR: 0.6 (0.3, 1.0) for one; 0.9 (0.5, 1.9) for two; 1.2 (0.6, 2.4) for 3+.
Conotruncal heart defects	Carmichael et al.	2003	American	Case-control	N=207	Frequency of drinking: less than once per week, once per week or more often. Frequency of drinking five or more drinks per occasion: never, less than once per week, once per week or more often. Periconceptional period.	Telephone interview about consumption during 1 month before conception to 3 months after conception	Maternal cigarette smoking, intake of multivitamin/mineral supplements containing folic acid, race-ethnicity and education level	OR= 1.2 (0.8, 1.7) for less than once per week; 1.6 (0.8, 3.0), for once per week or more often. 1.4 (0.7, 2.9) for drinking five or more drinks per occasion less than once per week; 1.7 (0.4, 7.0) for once per week or more often.

Conotruncal heart defects	Harvey et al.	2022	American	Cohort	N= 16 953	Alcohol related diagnoses code: yes or no.	Hospital discharge records from the California Office of Statewide Health Planning and Development and linked birth certificate records and interviews alcohol consumption during pregnancy.	Race and ethnicity, age at delivery, education, parity, and payer for delivery and preexisting diabetes, non-alcohol substance-related code during pregnancy, and mental health diagnosis complicating pregnancy.	Risk Ratios: Any non-critical congenital heart defect, 1.3 (1.2, 1.4); any critical congenital heart defect 1.5 (1.3, 1.8) for alcohol related diagnoses code.

OR=odds ratio; CI=confidence interval

[a]The values from the text and the abstract are different from the ones given in the table. Text value was included in the table above.

Supp. Table S2. Studies of prenatal alcohol exposure and the risk of urinary system defects

Birth defect	Author	Year	Study population	Study design	Number of cases	Prenatal Alcohol Exposure	Prenatal alcohol exposure measurement	Covariates	Findings
Renal or urinary anomalies	McDonald et al.	1992	American	Case-control	N=57	Seven or more drinks per week	Survey of occupational factors in pregnancy conducted about alcohol consumption during the 1st trimester of pregnancy.	Maternal age, educational level, and ethnic (color and language), cigarette and coffee consumption	OR = 0.9 (0.7, 1.2), for 1-2 drinks per week; OR= 1.0 (0.6, 1.5), for 3 to 6 drinks per week; OR= 1.2 (0.7, 2.2) for 7< per week.
Renal anomalies	Moore et al.	1997	American	Case-control	N=148	Light: <3 drinks per week. Moderate: 3 to 13 drinks per week. Heavy: 14 or more drinks per week.	Self-reported maternal alcohol consumption during the period from 1 month before through 3 months after conception. Maternal telephone interviews.	Maternal age, education, and race; mothers smoked, had diabetes mellitus, or used vitamins between 3 months before and the first 3 months after conception; and period of birth.	All renal anomalies: OR=0.8 (0.5, 1.2), for <3 drinks/week; 1.5 (1.0, 2.3) for alcohol intake 3-13 drinks/week and 1.7 (0.4, 6.0) for 14+ drinks/week. Agenesis/ hypoplasia: ORs= 0.5 (0.2, 1.4) for < 3 drinks/week; 2.5 (1.2, 5.1) for 3-13 drinks/week and 0.0 Indeterminant, for 14+ drinks/week. Hydroureteronephrosis: OR= 0.5 (0.2, 1.0) for < 3 drinks/week; 1.0 (0.5, 2.1) for 3-13 drinks/week and 2.3

									(0.4, 10.1) for +14 drinks/week. Duplication: OR=1.1 (0.3, 4.0) for <3 drinks/week; 2.1 (0.5, 8.0) for 3-13 drinks/week and 0.0 Indeterminant for +14 drinks/week. Multicycle dysplasia: OR=2.4 (1.0, .6) for < 3 drinks/week; 1.0 (0.2, 3.9) for 3-13 drinks/week and 4.1 (0.2, 32.1) for 14+ drinks/week.
Renal agenesis	Parikh et al.	2002	American	Case-control	N=188	Drinks per week during pregnancy. Per week was 1.5 in the case and 1.0 in the control group	Colorado State Birth Registry about alcohol consumption during pregnancy	Maternal race, age, education and preexisting diabetes.	OR=2.3 (1.0, 5.4)
Renal anomalies	Martinez-Frias et al.	2004	Spanish	Case-control	N=146	Type of alcoholic beverages, the amount per day (or other specified periodicity), and the moments of pregnancy. Standard drink of beer or wine = 10 gm and of distilled spirits has 20 gm	Interview the mothers within the first three days after delivery about alcohol consumption during all the gestational period	Birth weight, length, and occipital-frontal circumference, smoke during pregnancy	OR=0.8 (0.6, 1.1) for <10-20gm sporadically during gestation. OR=0.3 (0.0, 1.8) for >90gm or sporadic binges during gestation. OR=1.3 (0.8, 1.9) for 16-48gm daily ingestion. OR=2.9 (0.3, 73.3) for 56-88gm daily range ingestion. No results, for > 92gm daily ingestion
Bilateral renal agenesis or hypoplasia	Slickers et al.	2008	American, data from National Birth Defect Prevention Study	Case-control	N=75	Number of days and drinks that occurred during each month, as well as the maximum number of drinks that occurred at 1 sitting during each month. Alcohol exposure was represented by a 3-category variable, subdivided into that	Maternal medical and exposure histories were collected retrospectively through structured, computer-assisted telephone interviews within 24 months of delivery. Alcohol data was asked considering the month prior to conception through the third month of pregnancy	Acid folic use. Maternal age, race/ethnicity, study center, pregnancy body mass, periconceptional smoke and caffeine exposure	OR=1.4 (0.7, 2.5) and 1.9 (0.9, 4.2) for non-binge drinking and binge drinking.

						occurring with and without binge drinking (defined as 5 drinks on one occasion			
Hydronephrosis	O'Leary et al.	2013	Australian	Cohort	N=1365	Maternal alcohol diagnoses during pregnancy: (1) mental and behavioral disorder (alcohol-related), (2) alcohol-related diseases with a 100% attributable fraction (e.g., alcoholic liver disease), and (3) other alcohol codes	Western Australian Data Linkage Unit, data collected from birth records. Information about all pregnancy	Maternal age and year of birth, maternal Aboriginal status, maternal illicit drug use and socioeconomic status	OR: 2.1 (1.3, 5.4) for maternal alcohol related diagnoses
Renal anomalies	Woud et al.	2016	Dutch	Case-control	N=562	Alcohol consumption: No - Any	An etiologic research into Genetic and Occupational/environmental Risk factors for Anomalies in children. Questionnaire data about lifestyle during pregnancy and medical records analysis. Alcohol intake information about 3 months before or during pregnancy	Year of childbirth	Any alcohol consumption OR= 1.0 (0.8, 1.2)
OR=odds ratio; CI=confidence interval									

**Suppl. Table S3.** Studies of prenatal alcohol exposure and the risk of oral clefts								
**Author**	**Year**	**Study population**	**Study design**	**Number of cases**	**Prenatal Alcohol Exposure**	**Prenatal alcohol exposure measurement**	**Covariates**	**Findings**
Werler et al.	1991	American/ Canadian	case-control study	n=1,464	Maximum number of drinks in any one day (maximum intensity), number of drinking days per week (average frequency), and number of drinks per drinking day (average intensity).	Slone Epidemiology Unit Birth Defects Data. Interviews 6 months after delivery about alcohol consumption during the first four lunar months of pregnancy	Geographic center, interview year and maternal age.	OR=1.8 (0.8-4.4) a AC heavy intake (5 or more drinks per day); OR=3.0 (1.1-8.5) CLP heavy intake.
McDonald et al.	1992	Canadian	case-control study	n=96	Seven or more drinks per week	Survey of occupational factors in pregnancy about alcohol consumption during the 1st trimester of pregnancy.	Maternal age, educational level, and ethnic (color and language), cigarette and coffee consumption	CL/P. OR = 1.42 (0.9-2.4), for 1-2 drinks per week; OR= 1.68 (0.9-3.3), for 3 to 6 drinks per week, ; OR= 1.06 (0.3-4.2) , for 7< per week.
Munger et al.	1996	American	case-control study	n=287	Drinks per month. 1-3 drinks/month; 4-10 drinks/month; >10 drinks/month.	Iowa Birth Defects Registry. Structured questionnaires employed through telephone interviews. Self-reported alcohol consumption about intake during the 3 months before their pregnancy during their pregnancy.	Household income, maternal education, smoking, vitamin use; gender of child, and year of birth.	CLP= ORs: 1. 5(0.9-2.4) for 1-3 drink/mo; 3.5 (0.8-15.4), for 4-10 drinks/month; 4.0 (1.1-15.1), for >10 drinks/month. CP= ORs: 1.0 (0.5-2.0), for 1-3 drinks/month; 3.1 (0.5-21.0), for 4-10 drinks/month; 1.8 (0.3-2.1), for > 10 drinks/month.
Shaw et al.	1999	American	case-control study	n=731	<Weekly, weekly: 1 to 4 drinking episodes, or daily: every day. Also drinks/occasion.	Telephone interviews about alcohol consumption 1 month before through 3 months after conception.	Maternal cigarette smoking, race, education, or vitamin use.	Isolated CLP: ORs=0.86 (0.49-1.5), for <1 occasion/week; 3.4 (1.1-9.7), for drinking ≥5 drinks/occasion weekly or more frequently; Multiple CLP: ORs= 0.91 (0.39-2.5), for <1 occasion/week; 4.6 (1.2-18.8) multiple cleft lip; drinking ≥5 drinks/occasion weekly or more frequently

Lorente et al.	2000	European (British, Italian, French, Dutch)	case-control study	n=161	Nondrinkers (<1 drink/day during the first trimester of pregnancy both during the week and on the weekend). Ex-drinkers > 1drink/day before pregnancy but who stopped or decreased to < 1drink/day during the first trimester of pregnancy. Weekly (< 70 g and 70 g or more)	Maternal interview employing standardized questionnaires after birth or abortion about alcohol consumption during the first trimester of pregnancy.	Socioeconomic status, center, maternal age, tobacco consumption yes or no and area of residence.	Ex drinkers: ORs= 1.70 (0.65-4.43), for CP and 0.73 (0.36- 1.45) for CLP. Alcohol consumption during the first trimester: ORs=2.28 (1.02 - 5.09) for CP and 1.10 (0.56- 2.17) for CLP. Total weekend consumption: ORs= 2.3 (1.0 - 5.2) for CP and 1.1 (0.5 - 2.1) for CLP. Total consumption during the week: ORs= 1.3 (0.4- 4.4), for CP and 0.6 (0.2- 2.0) for CLP.
Beaty et al.	2001	American	case-control study	n=171	Any alcohol use during the first trimester	Telephone interview to mothers from the Maryland Birth Defects Reporting and Information System about alcohol consumption during the first 3 months of pregnancy.	Maternal education and age	ORs for any alcohol use= 0.40 (0.21–0.76), for CL/P; 0.53 (0.23–1.20), for CP.
Meyer et al.	2003	American	case-control study	n=912	Average weekly consumption (1, 1–2.9, and 3); average number of drinks per drinking day (1, 1–1.9, 2–2.9, and 3); and the maximum number of drinks consumed in a given day (1; 1–2; 3–4 and 5).	Maternal reports of alcohol consumption during the first 4 months of pregnancy from the Slone Birth Defect Study	Geographic center, interview year, maternal age, race, education and smoking during pregnancy	ORs average weekly consumption 1–2.9= 1.1 (0.8–1.4) and 3= 0.9 (0.7–1.4), for CL/P. 1-2.9= 1.1 (0.7–1.6) and 3=1.1 (0.7–1.7), for CLP. 1-2.9= 1.0 (0.7–1.6) and 3=0.8 (0.4–1.4), for CL. 1.-2.9= 0.8 (0.4–1.4) and 3=1.0 (0.6–1.9), for CP. ORs drinks per day 1-1.9= 1.1 (0.8–1.4); 2-2.9 = 1.0 (0.7–1.5) and 3= 1.0 (0.6–1.7), for CL/P. 1-1.9=1.1 (0.8–1.5); 2-2.9=1.2 (0.7–1.9) and 3= 1.2 (0.7–2.2), for CLP.1-1.9= 1.1 (0.7–1.5); 2-2.9= 0.8 (0.4–1.5) and 3= 0.8 (0.3–1.8), for CL. 1-1.9=0.6 (0.4–1.1); 2-2.9= 0.9 (0.4–1.8) and 3= 0.9 (0.4–2.1), for CP. ORs for maximum

								number of drinks per day: 1-2= 1.0 (0.8–1.3); 3-4= 1.1 (0.8–1.5) and 5=0.7 (0.5–1.1), for CL/P. 1-2=1.0 (0.8–1.4), 3-4= 1.4 (0.9–2.0) and 5= 0.7 (0.4–1.2), for CLP. 1-2=0.9 (0.7–1.4): 3-4= 0.7 (0.4–1.1) and 5= 0.7 (0.4–1.4), for CL. 1-2=0.9 (0.6–1.5); 3-4= 0.7 (0.4–1.3) and 5=0.9 (0.5–1.9), for CP.
Martinez-Frias et al.	2004	Spanish	case-control study	n=250	Type of alcoholic beverages, the amount per day (or other specified periodicity), and the moments of pregnancy. Standard drink of beer or wine = 10 gm and of distilled spirits has 20 gm.	Interview the mothers within the first three days after delivery during all pregnancy.	Birth weight, length, and occipital-frontal circumference, smoke during pregnancy	OR=0.93 (0.68-1.25), for <10-20gm sporadically during gestation. OR=3.49 (0.67-24.29), for >90gm or sporadic binges during gestation. OR=1.24 (0.94-1.63), for 16-48gm daily ingestion. OR=1.59 (0.47-5.6), for 56-88gm daily range ingestion. OR=4.48 (0.91-30.06), for > 92gm daily ingestion.
Chevrier et al.	2005	French	case-control study	n=240	First trimester of pregnancy distinguishing mothers who drank during the month before conception and stopped at the beginning of pregnancy, from mothers who never stopped their alcohol intake.	Maternal interview with a standardized questionnaire about alcohol consumption during first trimester of pregnancy.	Center, maternal age, ethnic origin, and maternal schooling, maternal smoking status and maternal dietary folate intake.	CL/P and CP. During the first trimester: OR=1.7 (1.0-2.9). During the preconceptional month: OR= 1.34 (0.8-2.3).
Romitti et al.	2007	American	case-control study	n=1,749	Monthly to weekly (1–4 drinks per month); weekly to every other day (5–15 drinks per month); every other day to daily (16–30 drinks per month); and daily with more than one drink per day (>30	Structured, computer-assisted telephone interviews about alcohol consumption of the period from 3 months before conception to the delivery date and data from National Birth Defects Prevention Study	Family history, maternal race/ethnicity, cigarette smoking, center, and duration of alcohol exposure	ORs: 1-4 drinks/month=1.2 (0.8-1.7); 5-15 drinks/month= 0.9 (0.5-1.4); 16-30 drinks/month= 0.4 (0.2-0.9) >30 drinks/month =1.0 (0.5-2.11), for CLP. 1-4 drinks/month =1.3 (1.0-1.9); 5-15 drinks/month= 1.1 (0.8-1.7); 16-30

					drinks per month).			drinks/month=1.1 (0.6-1.8); >30 drinks/month 1.1 (0.6-2.2), for CP.
Bille et al.	2007	Danish	case-control study	n=220	Alcohol consumption: yes/no. Units/week (0–7units).	Computer assisted telephone interviews about alcohol consumption during the first trimester of pregnancy. Danish National Birth Cohort	Parental age and social class	Alcohol consumption, OR: 1.11(0.79-1.55), for AC. OR: 1.11 (0.75-1.64) for CL and 1.10 (0.62-1.95) for CP. Units/week (0–7units) OR: 1.02 (0.88- 1.19) for AC; 0.99 (0.83-1.17) for CL/P and 1.10 (0.87-1.40) for CP. Units/week 1-2 OR= 1.06 (0.74-1.50) for AC; 1.05 (0.70-1.59) for CL/P and 1.6 (0.59-1.92) for CP. Units/week +3: 1.43 (0.74-2.79) for AC; 1.48 (0.68-3.19) for CL/P and 1.36 (0.45-4.15) for CP.
DeRoo et al.	2008	Norwegian	case-control study	n=573	Total number of drinks; number of drinks per day; average number of drinks per sitting; binge drinking episodes (5 drinks or more per sitting)	First-trimester alcohol consumption in self-administered questionnaires	Child's year of birth, mother's age group, prenatal smoking, education, household income, and family history of clefts	Binge drinking: OR=2.2 (1.1-4.2) for CLP and 2.6 (1.2-5.6) for CP. More than 3 binge drinking episodes: OR=3.2 (1.0, 10.2) for CLP, and 3.0 (0.7, 13.0) for CP.
Pei et al.	2015	Chinese	cross-sectional	n=29098	Alcohol intake frequency during pregnancy: No, <1/week, 1/week.	Structured questionnaire and medical records from local Hospitals about alcohol during pregnancy.	Infant gender, fetal number, residence during the pregnancy, mother's education, mother's age, and parity.	Prevalence rate ratios for AC: 9.02 (2.08-39.10), for <1 drink/week
Leite et al.	2009	Brazilian	case-control study	n=274	Alcohol consumption: the year before the conception, the first trimester of pregnancy. Amount (g) of	Interview to mother about information of consumption during the year previous of gestation and the first trimester of pregnancy.	Maternal age, schooling and smoking	1st trimester of gestation: ORs=2.08 (1.27-3.41) for CLP; 2.89 (1.25-8.3) for CP. Year previous of gestation: ORs= 1.80 (1.40-2.84)

					alcohol/day > than 96 g.			for CLP and 3.87 (1.73-8.65) for CP.
Sato et al.	2021	Japanese	cohort	n= 94,174	Non-drinker during pregnancy, former drinker who quit after pregnancy, and current drinker	Maternal alcohol consumption during pregnancy was obtained using the Food Frequency Questionnaire at 27 weeks of pregnancy about alcohol consumption during pregnancy.	Maternal age, sex of the baby, birth order, maternal educational attainment, annual household income, psychological distress, maternal active and passive smoking, body mass index, and folic acid	OR: Former drinkers = 0.87 (0.63-1.22); Current drinkers = 0.71 (0.22-2.27).
Kurita et al.	2021	Japanese	cohort	n=163	Non-drinkers: no alcohol consumption and/or quit drinking before pregnancy. Early drinkers: quit drinking during early pregnancy. Currently drinking: until the 2nd and 3rd trimester. Low (<1.5 drinks/week) and high (1.5+ drinks/week) absolute alcohol amount. 1 standard drink: 14g	Self-reported questionnaires during the 2nd and 3rd gestational trimester and data from the Japan Environment and Children's Study	Maternal age, smoking habit of the mothers, mother medical and obstetric history, maternal body mass before pregnancy, pregnancy complications,	OR= 0.84 (0.61–1.15), for quit drinking during early pregnancy; 0.82 (0.30–2.25), for current drinker.
[AC]= all clefts; CLP=cleft lip with or without cleft palate; CP=cleft palate; OR=odds ratio; CI=confidence interval								
a the values from the text and abstract are different from the ones given in the table, text value was included in the table above								

**Suppl. Table S4.** Studies of prenatal alcohol exposure and the risk of gastrointestinal system birth defects									
**Birth defect**	**Author**	**Year**	**Study population**	**Study design**	**Number of cases**	**Prenatal Alcohol Exposure**	**Prenatal alcohol exposure measurement**	**Covariates**	**Findings**

esophageal atresia	Wong-Gibbons et al.	2008	American, Data from National Birth Defect Prevention Study	case-control study	n=334	Month(s), average number of drinking days per month, average number of drinks per drinking day , and maximum number of drinks on one occasion per drinking month	Structured, computer-assisted telephone interviews about alcohol intake during the 3 months prior to conception and the duration of the pregnancy.	Plurality, maternal age, race and ethnicity, education, pre-pregnancy diabetes, infertility treatments, cigarette smoking (yes/no), duration of alcohol consumption, and study center	Any periconceptional exposure OR = 1.2 (0.8, 1.8)
esophageal atresia	Feng et al.	2016	Chinese	case-control study	n=130, controls: healthy children	Any alcohol consumption and binge drinking	Face-to-face questionnaires referred to the period from 1 month before conception to the end of first trimester of pregnancy	Maternal age, parity, gravidity, maternal education status, and employment status	ORs for any maternal alcohol intake: 0.91(0.41–2) and 2.63 (1.05-6.6) for maternal binge drinking
intestinal atresia/anal atresia/TEF	Martinez-Frias et al.	2004	Spanish	case-control study	n=112	Type of alcoholic beverages, the amount per day (or other specified periodicity), and the moments of pregnancy. Standard drink of beer or wine = 10 gm and of distilled spirits has 20 gm.	Interview the mothers within the first three days after delivery about alcohol during pregnancy	Birth weight, length, and occipital-frontal circumference, smoke during pregnancy	OR=1.19 (0.77-1.84), for <10-20gm sporadically during gestation. OR=1.34 (-0.25-7.55), for >90gm or sporadic binges during gestation. OR=0.89 (0.57-1.37), for 16-48gm daily ingestion. OR=1.67 (0.35-8.86), for 56-88gm daily range ingestion. OR= 3.01 (0.55-21.65), for > 92gm daily ingestion.

gastrointestinal obstruction	Kurita et al.	2021	Japanese	cohort	n=47	Non-drinkers: no alcohol consumption and/or quit drinking before pregnancy. Early drinkers: quit drinking during early pregnancy. Currently drinking: until the 2nd and 3rd trimester. Low (<1.5 drinks/week) and high (1.5+ drinks/week) absolute alcohol amount. 1 standard drink: 14g	Self-reported questionnaires during the 2nd and 3rd gestational trimester and data from the Japan Environment and Children's Study	Maternal age, smoking habit of the mothers, mother medical and obstetric history, maternal body mass before pregnancy, pregnancy complications,	OR= 1.33 (0.74–2.40), for quit drinking during early pregnancy; 1.75 (0.40–7.59), for current drinker.
OR=odds ratio; CI=confidence interval; TEF- tracheoesophageal fistula									

Suppl. Table S5. Studies of prenatal alcohol exposure and the risk herniation birth defects

**Birth defect**	**Author**	**Year**	**Study population**	**Study design**	**Number of cases**	**Prenatal Alcohol Exposure**	**Prenatal alcohol exposure measurement**	**Covariates**	**Findings**
diaphragmatic hernia	Caspers et al.	2010	American, data from National Birth Defects Prevention Study	case-control study	n=503	Alcohol exposure (yes/no), quantity, frequency, binge (yes/no) and variability of consumption.	Telephone interviews monthly for the 3 months prior to pregnancy, the first 3 months of pregnancy, and by trimester for months 4–6 and 7–9 of pregnancy	Infant sex, gestational age, maternal age, race and ethnicity, periconceptional cigarette smoking (yes/no), first- or second-degree family history, and study center	ORs= 1.1 (0.9–1.4), for any alcohol consumption. 0.9 (0.7–1.1), for 1 or more binge drinking episode. 0.9 (0.7–1.1), for 1-15 drinks/month; 1.1 (0.7–1.7) , for 16-30 drinks/month; 0.7 (0.4–1.4), for > 30 drinks/month
diaphragmatic hernia	McAteer et al.	2014	American	case-control study	n=492	Maternal use of alcohol: any use of alcohol in the specified period.	Information using birth records and seeing alcohol intake yes/no during pregnancy	Infant gender, maternal age, maternal race, maternal marital status, maternal parity, and maternal body mass index	OR=3.56 (1.36-9.83)

diaphragmatic hernia	Finn et al.	2022	American	case-control study	n=831	Alcohol (yes, no); average number of drinks per month, frequency, duration, binge drinking	Maternal interview reports about alcohol consumption during 3 months before and during pregnancy	Maternal early pregnancy smoking exposure, and study site is entered as a random intercept	ORs= 0.9 (0.8-1.1), for any alcohol consumption; 0.9 (0.8-1.1), for 1-15 drinks/month; 0.8 (0.6-1.2), for 16-30 drinks/month; 0.9 (0.6-1.5) >30 drinks/month; 0.9 (0.7-1.1), for >1 binge drinking episodes
gastroschisis	Werler et al.	1992	American, data from Slone Birth Defect Study	case-control study	n=76	Drinking days/ week, and average and maximum number of drinks in a drinking day	In person structured interview focused on maternal alcohol exposure during pregnancy about the first trimester of pregnancy	Age	RR= 1.8 (0.8-3.7) ; for 1-5 drinks /week; 2.9 (1.1-7.4), for >6 drinks/week; 0.8 (0.4-1.5), for 1-4 drinks/anytime ; 3.2 (1.5-6.7), for >5 drinks/any time
gastroschisis	Bird et al.	2009	American, data from National Birth Defects Prevention Study	case-control study	n=485	Any alcohol consumption: yes/no	Standardized computer-assisted interview about alcohol consumption during the exposure period was limited to 1 month before conception through 3 months postconception.	Study center, folic acid supplementation, body mass index, and preexisting and gestational diabetes, sex, maternal race, family income, parity, maternal age, any fever, smoking, marijuana, antibiotics consumption.	OR=1.38 (1.06-1.79)
gastroschisis	Richardson et al.	2011	American	case-control study	n=720	Alcohol yes or no. Binge drinking >4 drinks/occasion yes or no. Maximum of average drinks/month.	Maternal interviews about periconceptional consumption of alcohol (1 month prepregnancy through the third pregnancy month), and by quantity-frequency, duration, and beverage type	Race, age, state of residence at time of infant's birth, and periconceptional smoking	OR=1.4 (1.17-1.67) any alcohol intake, OR=1.3 (1.02-1.64)alcohol intake 1-4 drinks/month; OR=1.51 (1.15-1.97) alcohol intake 5-15 drinks/month; OR=1.89 (1.29-2.75) alcohol intake >30 drinks/month; OR=1.27 (1.01-1.59) drinking, not binge; OR=1.53 (1.21-1.92) binge drinking

									>- 4 drinks/occasion.
gastroschisis	Paranjothy et al.	2012	British	case-control study	n=91	Any alcohol consumption or binge drinking (≥ 6 units in one sitting/day)	Face to face questionnaire interviews about alcohol intake during the first twelve weeks of pregnancy	Socioeconomic level, cigarette consumption, portion of vegetables	ORs= >14 alcohol units 2.00 (0.6-7.1); 1.5 (0.6-3.5) binge drinking
gastroschisis	Palmer et al.	2013	British	case-control study	n=91	Binge drinking (≥ 6 units in one sitting/day)	Face to face questionnaire interviews about alcohol intake during the first twelve weeks of pregnancy	Maternal age, stressful situations, social support, marital status, change of address, socio economic situation, smoking, body mass index, caffeine and acid folic intake, vegetables diet, nausea synthoms	OR=1.6 (0.6-4.2)
gastroschisis	Rittler et al.	2015	South-American	case-control study	n=472	Alcohol yes or no	Interviews about alcohol consumption during any time in pregnancy	Hospital and year of birth	OR= 1.53 (0.97-2.43)
gastroschisis	Robledo-Aceves et al.	2015	Mexican	case-control study	n=90	Alcohol consumption yes or no	Questionnaires about alcohol consumption during the periconceptional period: 3 months before and 3 months after conception.	Maternal age, anemia during pregnancy, pre-pregnancy BMI <18.5 kg/m2, first-trimester tobacco smoking, and passive tobacco smoking	OR=3.4 (1.6-7.3), for any alcohol use during the first trimester of pregnancy.

gastroschisis	Werler et al.	2018	American	case-control study	n=1261	Alcohol: yes or no	Standardized computer-assisted questionnaire about alcohol consumption during the month before conception and the first trimester of pregnancy	Maternal age, Fever, Genitourinary Infect, Antiherpetic Use, Injury, Bronchodilator Use, Opioid Use, Smoking, Illicit Drug Use, Oral Contraceptive Use, Interpregnancy interval <12 mos, Residential Move, Aspirin, Ibuprofen, Venlafaxine, Paroxetine	OR=1.3 (1.1, 1.5)
gastroschisis	Dewberry et al.	2020	American	case-control study	n=236	Alcohol yes or no	Interview about alcohol consumption during the prepregnancy period and the first trimester of pregnancy	Smoking and maternal age	OR = 2.37 (1.27, 4.43), for prenatal alcohol consumption; 15. 1 (4.21-54.1) for first trimester.
omphalocoele	Bird et al.	2009	American, data from National Birth Defects Prevention Study	case-control study	n=168	Any alcohol consumption: yes/no	Standardized computer-assisted interview about alcohol consumption during the exposure period was limited to 1 month before conception through 3 months postconception.	Study center, folic acid supplementation, body mass index, and preexisting and gestational diabetes, sex, maternal race, family income, parity, maternal age, any fever, smoking, marijuana, antibiotics consumption.	OR=1.53 (1.04-2.25)

omphalocoele	Richardson et al.	2011	American, data from National Birth Defects Prevention Study	case-control study	n=254	Alcohol yes or no. Binge drinking >4 drinks/occasion yes or no. Maximum of average drinks/month.	Maternal interviews about periconceptional consumption of alcohol (1 month prepregnancy through the third pregnancy month), and by quantity-frequency, duration, and beverage type	Race, age, and state of residence at time of infant's birth.	OR=1.5 (1.15-1.96) any alcohol intake; OR=1.55 (1.12-2.14) alcohol intake 1-4 drinks/month; OR=1.73 (1.05-2.87) alcohol intake16-30 drinks/month; OR=1.43 (1.06-1.93) drinking, no binge; OR=1.71 (1.19-2.45) binge drinking ≥4/occasion;
OR=odds ratio; CI=confidence interval; RR=relative risk									

Suppl. Table S6. Studies of prenatal alcohol exposure and the risk of skeletal system and spine defects

**Birth defect**	**Author**	**Year**	**Study population**	**Study design**	**Number of cases**	**Prenatal Alcohol Exposure**	**Prenatal alcohol exposure measurement**	**Covariates**	**Findings**
clubfoot	Werler et al.	2014	American	case-control	n=646	No drinkers <2 drinks/day. Drinkers>2 drinks/day. Low ≤3 drinks/day High >3.0 drinks/day	Telephone interview using a computerized, standardized questionnaire about their consumption 1 month before the last menstrual period (LMP) to delivery	All exposures, study center, child sex, and maternal race/ethnicity, parity, obesity, fertility treatment, and LM 2–4 uses of opioids, selective serotonin reuptake inhibitor, phenergan, ondansetron, pseudoephedrine, diphenhydramine, amoxicillin, and salicylates.	Anytime from the last menstrual period 2-4 OR: ≤3/day =1.09 (0.77, 1.54) and >3/day= 1.25 (0.80, 1.95)

spina bifida	Benedum et al.	2013	American	case-control	n=776	Heavy drinker: ≥4 drinks/day. Frequency: Days/week: <1, 1, 2, and ≥3. Intensity: Drinks/drinking day (<1, 1, 2, and ≥3) at any time during the first lunar month after last month period.	Maternal telephone interviews about alcohol consumption during 2 months prior to conception and the first month of pregnancy	Period of evaluation: 1988-1997 adjusted for maternal education; and period 1998+ adjusted for NSAID use and folic acid antagonist medication use	ORs for the period 1988-1997, Heavy drinking: 1.1 (0.7-1.6) and for the year 1998+ heavy drinking: 1.2 (0.8-2.0), during the first month of pregnancy.
spina bifida	Makelarski et al.	2013	American	case-control	n=703	Exposed: drinking alcohol during one or more periconceptional months. Monthly to weekly (1–4 drinks per month); weekly to every other day (5–15 drinks per month); every other day to daily (16–30 drinks per month); and daily with more than one drink per day (>30 drinks per month). Binge or not binge.	Maternal interview about alcohol consumption during the periconceptional period included the month before conception (B1) and the two months postconception (P1, P2)	Maternal race/ethnicity, education, pre-pregnancy body mass index, cigarette smoking, and site	ORs drinks/month: 1-4= 1.0 (0.8-1.3); 5-15= 1.0 (0.8-1.3); 16-30= 0.7 (0.5-1.2); >30= 1.0 (0.6- 1.7). OR for one or more binge drinking episodes= 1.0 (0.8-1.4).
neural tube defect	McDonald et al.	1992	American/Canadian	case-control	n=190	Seven or more drinks per week	Survey to current and previous pregnancies of more than 20 weeks' duration about alcohol consumption during the first trimester of pregnancy.	Maternal age, educational level, and ethnic (color and language), cigarette and coffee consumption	OR = 0.91 (0.7-1.2), for 1-2 drinks per week; OR= 0.96 (0.6-1.5), for 3 to 6 drinks per week, ; OR= 1.24 (0.7-2.2) , for 7< per week.

neural tube defect	Suarez et al.	2008	American	case-control	n=175	Alcohol use in the preconception period: <1 drink daily or > 1 drink daily. First trimester: <1 drink daily or > 1 drink daily. Binge drinking: 3 or fewer drinks on any occasion or >3 drinks on any occasion.	In person interviews about alcohol consumption during 3 months before and 3 months after conception to Mexican American women	Maternal age, education, body mass index, and folate intake	ORs for the preconception period: <1 drink/day = 1.3 (0.8-2.2); >1 drink/day= 1.7 (0.6-4.6). ORs for first trimester <1 drink/day= 1.2 (0.6-2.2); >1 drink/day= 1.5 (0.4-6.1).ORs for binge drinking: <3 drinks/occasion= 1.0 (0.4-2.7); >3 drinks/occasion= 1.7 (0.8-3.6)
neural tube defect	Louden et al.	2020	American	case-control	n= 1,922	Using a 30-day month categorization: monthly to weekly (1–4 drinks per month); weekly to every other day (5–15 drinks per month); every other day to daily (16–30 drinks per month); and daily with more than one drink per day (>30 drinks per month). Binge drinking: no consumption; consumption without any binge episodes; or one or more binge episodes	Interviews about alcohol consumption any time during the 3 months before conception through the end of pregnancy	NBDPS site	OR for any alcohol: 0.8 (0.7-0.9). OR for average number of drinks/month: 1-4= 0.8 (0.7-0.9); 5-15= 0.8 (0.7-0.9); 16-30= 0.6 (0.5-0.8); >30= 0.8 (0.6-1.1). OR for one or more binge drinking episodes= 0.8 (0.7-1.0)
OR=odds ratio; CI=confidence interval									

Suppl. Table S7. Studies of prenatal alcohol exposure and the risk of genital birth defects									
**Birth defect**	**Author**	**Year**	**Study population**	**Study design**	**Number of cases**	**Prenatal Alcohol Exposure**	**Prenatal Alcohol Exposure measurement**	**Covariates**	**Findings**

cryptorchidism	Carbone et al.	2007	Italian	case-control	n=48	Alcohol use during pregnancy: 1-7 glasses/week and ≥8 glasses/week	In person maternal questionnaire and interview about alcohol consumption from 3 months prior to 3 months after conception	Mother's age, Mother's education, parity, birth weight, time to pregnancy, condom use, use of anti-abortion drug, mother's gynecological diseases, and father's urogenital diseases	OR=0.99 (0.40–2.44), for alcohol intake 1-7 glasses/week; OR=4.55 (1.23-16.79), for alcohol consumption ≥8 glasses/week
cryptorchidism	Damgaard et al.	2007	Danish-Finish	cohort	n=1,042	Average number of glasses of wine per week, bottles of beer per week, liquor glasses of spirits per week: 1>/week, the precise number of drinks/week, binge/week or do not know/do not wish to answer	Questionnaire by mail late in the first or early in the second trimester of pregnancy. Women were instructed to complete the questionnaire at the beginning of the third trimester and to return it by mail before birth, about alcohol consumption during the hole pregnancy	Country, smoking, caffeine intake, maternal age, social class, parity, maturity, birth weight, and binge episodes and alcoholic drinks/week mutually	OR=1.17 (1.03-1.51) alcohol drinking as continuous variable; ORs=0.94 (0.58–1.51) for >1 drinks/week; 1.28 (0.72–2.27), for >2 drinks/week; 1.21 (0.55–2.66), for >3 drinks/week; 1.77 (0.67–4.69), for >4 drinks/week; 3.10 (1.05-9.10), for > 5 drinks/week; 5.47 (1.59–18.88), for > 6 drinks/week; 6.54 (1.56–27.43), for >7 drinks/week; 16.78 (3.48–81.02), for > 8 drinks/week; 31.89 (3.96–256.93), for > 9 drinks/week. OR=1.18 (0.77–1.83), for

									binge during pregnancy
cryptorchidism	Jensen et al.	200 7	Danish	case-control	n=270 (185)[a]	Weekly intake of beer, wine and spirits, as well as on the number of binge episodes (≥8 units of alcohol on one occasion) during the pregnancy	Pregnant women around the 36th week of gestation, filled in a comprehensi ve self-completed questionnaire about alcohol consumption during pregnancy	Maternal and paternal age at delivery, time to index pregnancy, infertility treatment, parity, socioecono mic group, mothers' daily smoking, birth weight, gestational age, placental weight, and binge episodes and weekly alcohol intake mutually	ORa= 0.8 (0.6–1.2), for 1-4 drinks/wee k; 0.5 (0.2–1.2), for 5-9 drinks/wee k; 0.6 (0.1–2.7), for >10 drinks/wee k. OR= 1.4 (0.9–2.1), for binge episodes.

cryptorchidism	Strandberg-Larsen et al.	2009	Danish	case-control	n=1.598 (398)[a]	The preconceptional period (weeks 1–2), the period of fertilization and implantation (weeks 3–4), the early embryonic period (weeks 5–6), the late embryonic period when the testes begins to form (weeks 7–9), the transabdominal migration of the tests (weeks 10–15) and the mid-fetal phase (weeks 16–25). Average drinking during pregnancy and binge episodes as 0, 1, 2 and 3+ in the period before each interview.	Two computer-assisted telephone interviews around gestational weeks 17 and 32 about alcohol consumption during the preconceptional period and different periods in gestation.	Maternal age, parity, time to pregnancy and infertility treatment, self-reported diabetes mellitus, smoking habits during pregnancy and occupational status in the household	HRs=1.82 (0.97-3.37) ≥3 binge drinking episodes in pregnancy; 0.99 (0.65-1.49), for 1/2 and 1 1/2 drinks/week; 0.98 (0.47-2.08), for 2 to 3 1/2 drinks/week. (First-born boys of mother who conceived within 6 months of trying and orchiopexy procedure)
cryptorchidism	Brouwers et al.	2010	Dutch	case-control	n=200	Alcoholic beverages during pregnancy	Questionnaires about lifestyle during pregnancy	Year of birth	OR=0.9 (0.5-1.5)
hypospadiasis	Carbone et al.	2007	Italian	case-control	n=43	Alcohol use during pregnancy: 1-7 glasses/week and ≥8 glasses/week	In person maternal questionnaire and interview about alcohol consumption from 3 months prior to 3 months after conception	Mother's age, Mother's education, parity, birth weight, time to pregnancy, condom use, use of anti-abortion drug, mother's gynecological diseases, and father's urogenital diseases	ORs= 1.05 (0.44–2.53), for 1-7 glass/week; 1.70 (0.30–9.52) for >8 glass/week

genital defects	Martinez-Frias et al.	2004	Spanish	case-control	n=575	Type of alcoholic beverages, the amount per day (or other specified periodicity), and the moments of pregnancy. Standard drink of beer or wine = 10 gm and of distilled spirits has 20 gm.	Interview the mothers within the first three days after delivery about alcohol consumption over the gestation	Birth weight, length, and occipital-frontal circumference, smoke during pregnancy	OR=0.95 (0.78-1.15), for <10-20gm sporadically during gestation. OR=0.69 (0.33-1.42), for >90gm or sporadic binges during gestation. OR=1.04 (0.88-1.24), for 16-48gm daily ingestion. OR=0.65 (0.24-1.72), for 56-88gm daily range ingestion. OR=2.61(0.63-12.4), for > 92gm daily ingestion.
male genital defects	Kurita et al.	2021	Japanese	cohort	n= 266	Non-drinkers: no alcohol consumption and/or quit drinking before pregnancy. Early drinkers: quit drinking during early pregnancy. Currently drinking: until the 2nd and 3rd trimester. Low (<1.5 drinks/week) and high (1.5+ drinks/week) absolute alcohol amount. 1 standard drink: 14g	Self-reported questionnaires during the 2nd and 3rd gestational trimester and data from the Japan Environment and Children's Study	Maternal age, smoking habit of the mothers, mother medical and obstetric history, maternal body mass before pregnancy, pregnancy complications,	OR= 0.88 (0.69–1.12), for quit drinking during early pregnancy; 0.93 (0.45–1.91) for current drinker.

OR=odds ratio; CI=confidence interval

[a] in brackets number of boys who underwent orchidopexy
